# Trends in broad-spectrum antibiotic prescribing for children with acute otitis media in the United States, 1998–2004

**DOI:** 10.1186/1471-2431-9-41

**Published:** 2009-06-24

**Authors:** Andrew S Coco, Michael A Horst, Angela S Gambler

**Affiliations:** 1 Research Institute & Department of Family Medicine, Lancaster General Hospital, North Duke Street, Lancaster, PA 17604-3555, USA

## Abstract

**Background:**

Overuse of broad-spectrum antibiotics is associated with antibiotic resistance. Acute otitis media (AOM) is responsible for a large proportion of antibiotics prescribed for US children. Rates of broad-spectrum antibiotic prescribing for AOM are unknown.

**Methods:**

Analysis of the National Ambulatory Medical Care Survey and National Hospital Ambulatory Medical Care Survey, 1998 to 2004 (N = 6,878). Setting is office-based physicians, hospital outpatient departments, and emergency departments. Patients are children aged 12 years and younger prescribed antibiotics for acute otitis media. Main outcome measure is percentage of broad-spectrum antibiotics, defined as amoxicillin/clavulanate, macrolides, cephalosporins and quinolones.

**Results:**

Broad-spectrum prescribing for acute otitis media increased from 34% of visits in 1998 to 45% of visits in 2004 (P < .001 for trend). The trend was primarily attributable to an increase in prescribing of amoxicillin/clavulanate (8% to 15%; P < .001 for trend) and macrolides (9% to 15%; P < .001 for trend). Prescribing remained stable for amoxicillin and cephalosporins while decreasing for narrow-spectrum agents (12% to 3%; P < .001 for trend) over the study period. Independent predictors of broad-spectrum antibiotic prescribing were ear pain, non-white race, public and other insurance (compared to private), hospital outpatient department setting, emergency department setting, and West region (compared to South and Midwest regions), each of which was associated with lower rates of broad-spectrum prescribing. Age and fever were not associated with prescribing choice.

**Conclusion:**

Prescribing of broad-spectrum antibiotics for acute otitis media has steadily increased from 1998 to 2004. Associations with non-clinical factors suggest potential for improvement in prescribing practice.

## Background

Use and overuse of antibiotics is associated with the development and spread of resistant bacteria [1]; a problem continuing to gain attention from national organizations as a significant threat to the public health [2]. It is important to avoid unnecessary pediatric antibiotic use because children represent a large reservoir for resistant organisms [3]. Acute otitis media (AOM) accounts for up to 40% to 50% of antibiotics prescribed for United States children less than 2 years of age [4]. Encouragingly, overall pediatric antibiotic prescribing has declined recently [5,6], and visits for AOM have decreased, apparently in conjunction with implementation of the pneumococcal conjugate vaccine (PCV)[4,7], although PCV only has a marginal effect on decreasing AOM episodes for an individual child [8]. Visit-rate prescribing of antibiotics for AOM, however, has not decreased [6].

Antibiotic treatment is recommended for most children with AOM in the United States [9,10] with therapy targeted primarily at Streptococcus pneumoniae, the most frequent, clinical pathogen [11,12]. Amoxicillin remains the recommended first-line therapy for uncomplicated cases [9,11], but as resistant strains of Streptococcus pneumoniae have increased considerably in the past 20 years [13-15] there is concern of increased prescribing of broad-spectrum agents. A counteracting factor to this concern is the effectiveness of PCV in reducing resistant strains of Streptococcus pneumoniae [16,17]. By providing reassurance that common pathogens are still sensitive to amoxicillin, PCV may mitigate antibiotic choices for broader coverage. It is important for the Centers for Disease Control and Prevention (CDC) and other national organizations involved in curtailing antibiotic resistance to gain a better understanding on how physicians have responded to these influences in their prescribing choices for AOM.

To measure changes in the rate of broad-spectrum and other types of antibiotics prescribed to children seen with a diagnosis of AOM, data was analyzed from the National Ambulatory Medical Care Survey (NAMCS) and National Hospital Ambulatory Medical Care Survey (NHAMCS) from 1998 to 2004. The NAMCS and NHAMCS are the only surveys of outpatient settings in the United States that collect prescribing information and produce unbiased national estimates. These data allow for a comprehensive assessment of the antibiotic prescribing for children with AOM on a national level over time.

## Methods

### Study Design and Administration

Data for this study was compiled from the 1998 – 2004 NAMCS and NHAMCS. The surveys are administered by the National Center for Health Statistics (NCHS) for the CDC. The NAMCS collects information on patient visits to non-federally funded, community, office-based physician practices in the United States. Federally qualified health centers and non-federal government clinics are included in the database. NAMCS has a three-tiered design based on geographic location, physician specialty and individual visits within the practice. The NCHS weighs each visit by taking into account practice location and physician specialty. Physicians are randomly selected from the master files of the American Medical Association and the American Osteopathic Association. Each physician is randomly assigned to a one-week reporting period. During this period, data for a systematic random sample of visits is recorded by the physician or office staff on a standardized encounter form provided for that purpose and checked for completeness by NCHS field staff. Physicians in the sample complete 30 records over a one week period.

The NHAMCS measures utilization and provision of ambulatory care services at US hospitals. Using a 4-stage probability sample design, NHAMCS collects a nationally representative sample of visits to hospital outpatient departments and emergency departments based in nonfederal general and short-stay hospitals. NHAMCS data is collected by hospital staff members at sampled hospitals and monitored by NHAMCS field representatives. Visit information is collected during a randomly assigned 4-week reporting period each year. The NCHS institutional review board approved the protocols for the NAMCS and NHAMCS, including a waiver of the requirement for informed consent. Further description of the NAMCS and NHAMCS methodology is available from the NCHS [18,19].

### Study Sample: Episodes of Care for Acute Otitis Media

Up to 3 diagnoses were recorded for each visit as free text and then coded using the International Classification of Diseases, Ninth Revision (ICD-9)[20]. Visits with the following diagnoses (ICD-9-CM diagnosis of 381.0 – acute nonsuppurative otitis media, 381.4 – nonsuppurative otitis media, not specified as acute or chronic, 382.0 – acute suppurative otitis media, 382.4 – unspecified suppurative otitis media, or 382.9 – unspecified otitis media) were included. Visits with an alternative diagnosis that could have justified an antibiotic prescription were excluded. These were visits with diagnoses such as acute sinusitis (ICD-9-CM 461), chronic sinusitis (ICD-9-CM 473), acute pharyngitis (ICD-9-CM 462), acute tonsillitis (ICD-9-CM 463), streptococcal sore throat (ICD-9-CM 034.0), or pneumonia (ICD-9-CM 481 – 486). Additionally, visits were recorded as being for an acute or chronic problem. To focus specifically on acute episodes of otitis media, visits coded as a chronic problem were excluded from the study sample.

### Covariates

Patient age up to 12 years old (collapsed to < 2 years, ≥ 2 years), sex, race (categories collapsed to white or non-white), and insurance status (categories collapsed to private, Medicare/Medicaid, or other) were recorded for each visit. Provider self-selected specialty (NAMCS) and clinic type (NHAMCS) were coded as pediatrics (includes pediatric clinic), family practice (includes general medical clinic) or otolaryngology (includes surgery clinic). Geographic region was recorded as well. Up to 3 "complaints, symptoms, or other reason(s) for visit" were abstracted as free text and then coded centrally using a standard reason for visit classification (RVC) system [18,19]. Visits with symptoms of ear pain (RCV code 13551) and fever (RCV code 10100) were identified.

### Outcome: Antibiotics Prescribed

Up to 6 medications (8 since 2003) were recorded for each visit. Medications were coded via an ambulatory care drug database coding system [21]. Antibiotics were identified by using the National Drug Code Directory class prefix 03 ("antimicrobials") and excluded polymyxins, aminoglycosides, antimycobacterial, antifungal, and antiviral agents. Topical agents including topical anti-infectives (drug class code 1271) and topical otics (drug class code 1670) were excluded. Amoxicillin was defined to include ampicillin. Narrow-spectrum agents other than amoxicillin were primarily trimethoprim/sulfamethoxazole or erythromycin compounds. Broad-spectrum agents were defined to include amoxicillin/clavulanate, cephalosporins, macrolides (except erythromycin), and quinolones. Visits with a quinolone prescription comprised only 1% of sample records and thus were not sufficient for a separate trend analysis. If more than 1 antibiotic was used in a single visit (4% of sample records), we counted each antibiotic prescribed in its respective subclass, but the visit only counted once as an episode of care in which an antibiotic was prescribed for the trend analyses. If a patient received both a broad-spectrum and a narrow-spectrum antibiotic, we considered the visit as one in which a broad-spectrum antibiotic was prescribed for that trend analysis.

### Data Analysis

Categorical variables were evaluated with the χ^2 ^test. Linear regression, with calendar year as a predictor variable, was used to analyze time trends. In order to control for potential confounding variables a multivariate logistic regression model was developed to determine associations with receipt of a broad-spectrum antibiotic prescription, while controlling for age, race, calendar year, insurance status, geographical region, visit setting, physician specialty, and symptoms of ear pain or fever. Population estimates were based on the survey weights that accounted for the complex survey design by using the svy command provided in Stata version 10 (StataCorp, College Station, Texas). All P values are 2-tailed; P < .05 was considered significant.

Repeat visits by the same individual are not accounted for because unique identifiers are not provided in the surveys. However, because data are drawn from 1 weeks' duration at a given office (NAMCS) or 4 weeks' duration at a given facility (NHAMCS), repeated visits are likely to be relatively uncommon in the accrued data.

## Results

### Antibiotic Prescribing Trends

**Figure 1:**
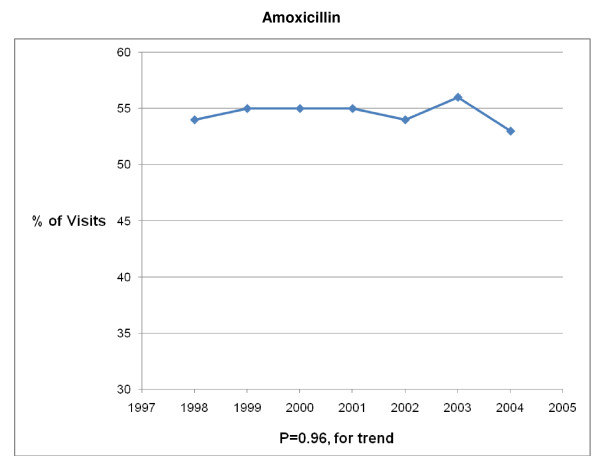
**Trends in antibiotic prescribing for children with acute otitis media in the United States, 1998–2004**. Based on 6878 visits in the National Ambulatory Medical Care Survey and National Hospital Ambulatory Care Survey.

**Figure 2:**
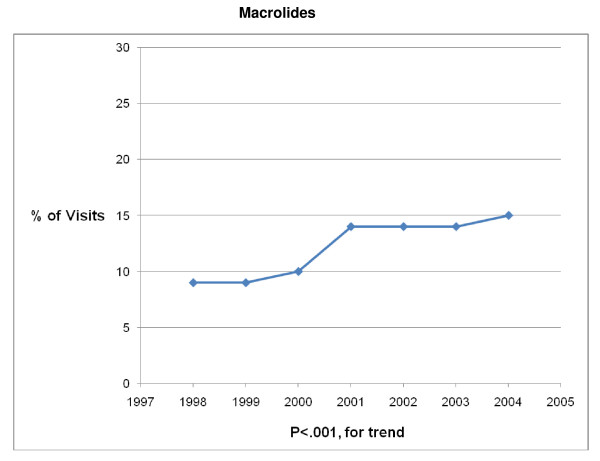
**Trends in Macrolides prescribing for children with acute otitis media in the United States, 1998–2004**. Based on 6878 visits in the National Ambulatory Medical Care Survey and National Hospital Ambulatory Care Survey.

**Figure 3:**
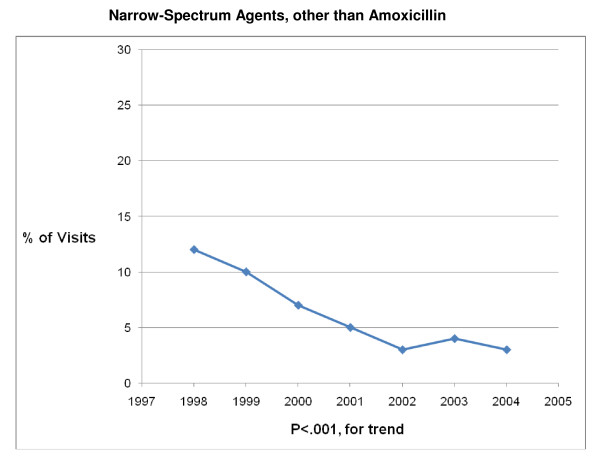
**Trends in Narrow-Spectrum Agents, other than Amoxicillin prescribing for children with acute otitis media in the United States, 1998–2004**. Based on 6878 visits in the National Ambulatory Medical Care Survey and National Hospital Ambulatory Care Survey.

**Figure 4:**
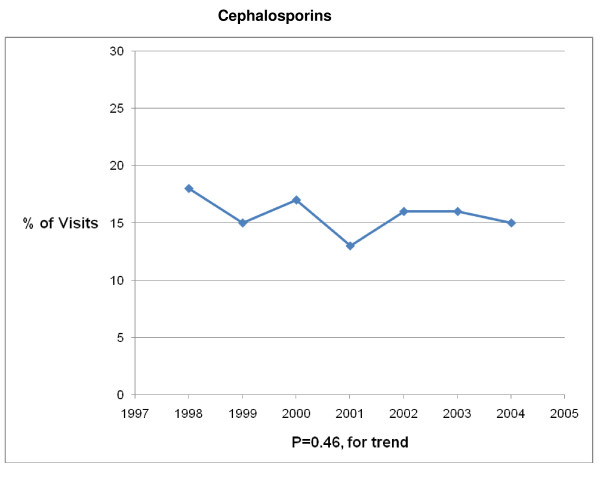
**Trends in Cephalosporins, other than Amoxicillin prescribing for children with acute otitis media in the United States, 1998–2004**. Based on 6878 visits in the National Ambulatory Medical Care Survey and National Hospital Ambulatory Care Survey.

**Figure 5:**
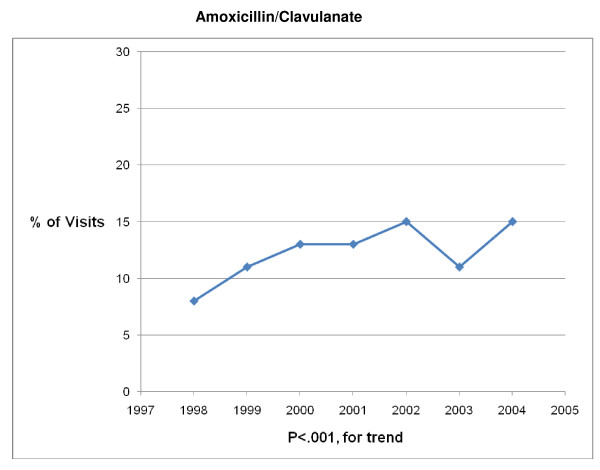
**Trends in Amoxicillin/Clavulanate prescribing for children with acute otitis media in the United States, 1998–2004**. Based on 6878 visits in the National Ambulatory Medical Care Survey and National Hospital Ambulatory Care Survey.

**Figure 6:**
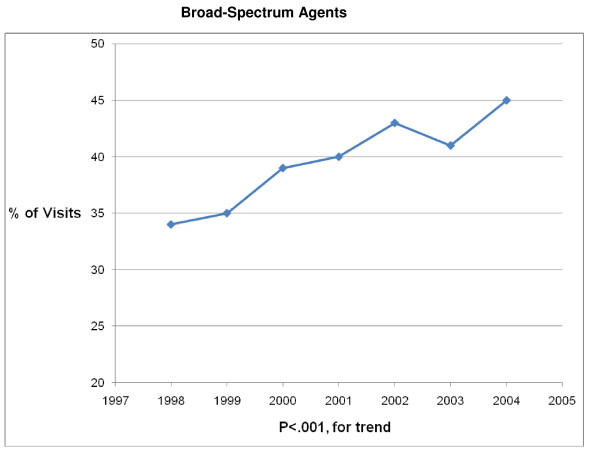
**Trends in Broad-Spectrum Agents prescribing for children with acute otitis media in the United States, 1998–2004**. Based on 6878 visits in the National Ambulatory Medical Care Survey and National Hospital Ambulatory Care Survey. Broad-spectrum antibiotics: amoxicillin/clavulanate, macrolides, cephalosporins and quinolones.

The 1998 – 2004 NAMCS and NHAMCS were conducted on encounters from 693,505 patient visit records. After excluding visits with a concomitant non-AOM, antibiotic-appropriate, diagnosis, 8325 records remained. Over the 7-year study period physicians prescribed antibiotics in 83% (6878/8325) of AOM visits. Broad-spectrum antibiotics were prescribed in 41% (2839/6878) and amoxicillin was prescribed in 56% (3880/6878) of visits in which an antibiotic was prescribed (Table 1). Broad-spectrum antibiotic prescribing increased from 34% of visits in 1998 to 45% of visits in 2004 (P < .001 for trend) (Figures 1, 2, 3, 4, 5 and 6). The broad-spectrum trend was attributable to an increase in the prescribing of amoxicillin/clavulanate (8% to 15%; P < .001 for trend), macrolides (9% to 15%; P < .001 for trend) (Figures 1, 2, 3, 4, 5 and 6). Cephalosporin (18% to 15%; P = .46 for trend) and amoxicillin prescribing (54% to 53%; P = .96 for trend) remained stable (Figures 1, 2, 3, 4, 5 and 6). Prescribing of narrow-spectrum agents other than amoxicillin decreased (12% to 3%; P < .001 for trend) over the study period (Figure 1, 2, 3, 4, 5 and 6).

**Table 1:** Types of antibiotics prescribed for children with acute otitis media in United States physician's offices, hospital outpatient departments and emergency departments 1998 to 2004 (N = 6878)

Antibiotic	Number of Visits	% of Visits
Narrow-spectrum agents		
Amoxicillin	3880*	56
Other narrow-spectrum agents	448^†^	7
Total	4328	63
		
Broad-spectrum agents		
Cephalosporins	1076	16
Amoxicillin/clavulanate	848	12
Macrolides	841	12
Quinolones	74	1
Total	2839^‡^	41
		
All antibiotics	7167	104^§^

Abbreviations: CI, confidence interval.

*Includes 129 visits in which both amoxicillin and a broad-spectrum agent were prescribed.

^†^Includes 26 visits in which both another narrow-spectrum agent and a broad-spectrum and were prescribed.

^‡^Two broad-spectrum agents were prescribed in 120 visits.

^§^More than one antibiotic was prescribed in 4% (285/6878) of visits.

In multivariable logistic regression modeling, the only independent positive predictor of broad-spectrum antibiotic prescribing was calendar year (odds ratio [OR], 1.07 per year; 95% CI, 1.05–1.10) (Table 2). The other independent predictors of broad-spectrum antibiotic prescribing; ear pain, non-white race, Medicare/Medicaid or other insurance (compared to private insurance), hospital outpatient department setting, emergency department setting, and West region (compared to South and Midwest regions), were each associated with lower rates of broad-spectrum prescribing. The other two clinical factors, aside from ear pain, associated with more severe infections – age and fever- were not independent predictors of broad-spectrum antibiotic prescribing.

**Table 2:** Predictors of broad-spectrum* antibiotic choice among pediatric visits with an antibiotic prescription in United States physician's offices, hospital outpatient departments, and emergency departments, 1998–2004

Characteristic	Proportion of Visits (%) (N = 6878)	Visits with a Broad-Spectrum Antibiotic Prescription (%)†	Adjusted OR (95% CI) of Broad-Spectrum Antibiotic Prescription
Calendar year, per 1 y	100	--	1.07 (1.05 – 1.10)
Sex			
Female	47	46	1.00
Male	53	54	1.02 (0.92 – 1.12)
Age			
< 2 years	49	48	1.00
≥ 2 years	51	52	1.05 (0.94 – 1.18)
Race			
White	73	78	1.00
Non-White	27	22	0.65 (0.58 – 0.73)
Insurance			
Private	43	47	1.00
Medicaid/Medicare	43	41	0.85 (0.76 – 0.95)
Self-pay & Other	14	12	0.76 (0.65 – 0.89)
Physician Specialty‡			
Pediatrics	60	59	1.00
Family Practice	37	37	1.07 (0.91 – 1.24)
Otolaryngology	3	4	1.47 (0.96 – 2.25)
Setting			
Physician Office	17	22	1.00
Hospital Clinic	29	29	0.77 (0.66 – 0.90)
Emergency Department	54	49	0.61 (0.53 – 0.71)
Region			
West	20	16	1.00
South	26	41	2.27 (1.97 – 2.62)
Midwest	33	25	1.38 (1.19 – 1.60)
Northeast	21	18	1.15 (0.98 – 1.35)
Fever			
No	62	63	1.00
Yes	38	37	0.96 (0.86 – 1.07)
Ear Pain			
No	68	70	1.00
Yes	32	30	0.83 (0.73 – 0.94)

Abbreviations: CI, confidence interval; OR, odds ratio.

*Broad-spectrum antibiotics: amoxicillin/clavulanate, macrolides, cephalosporins and quinolones.

†As a percentage of the total number of patients prescribed an antibiotic.

‡Physician office and hospital outpatient department settings only (N = 3168).

### Population Visit Rates

This sample represents an estimated 90 million (95% confidence interval [CI], 83 million-97 million) visits in the US by children aged 12 years and younger with AOM to pediatricians, other primary care physicians and clinics, and emergency departments between 1998 and 2004. Annual visits for AOM averaged 12.8 million visits per year, ranging from 15.2 million visits in 1998 to 9.3 million visits in 2004. There was a significant change in the proportion of all visits for children 12 years and younger diagnosed with AOM over time, decreasing from 10.3% of visits in 1998 to 6.8% in 2004 (P < .001 for trend).

## Discussion

Although amoxicillin remained the recommended antibiotic for AOM and PCV had become widely implemented during the study period, we found that among children receiving antibiotics, broad-spectrum prescribing steadily increased from 34% of visits in 1998 to 45% of visits in 2004, a 32% increase over the 7 year interval. An 88% increase in amoxicillin/clavulanate prescribing and a 67% increase in macrolide prescribing were largely responsible for the progressive trend in broad-spectrum usage. So although fewer children were being diagnosed with AOM, those that presented for care and received an antibiotic prescription had a high likelihood of receiving a broad-spectrum agent by 2004.

Other studies have looked at the types of antibiotics prescribed for AOM. Broad-spectrum antimicrobials were used to treat 28% of uncomplicated new AOM infections in a sample of visits to community pediatricians in 1999–2000 [22]. More recently, it was found that for children with AOM less than 2 years of age, 66% received penicillins, 21% cephalosporins, and 11% macrolides including erythromycin [4]. Amoxicillin/clavulanate prescriptions were not reported separately. Curiously, in that study, antibiotics were prescribed in only 58% of visits related to AOM. Nonetheless, it is difficult to make comparisons between these results and our analysis which is derived from a nationally representative sample of visits over a 7 year period. The increased prescribing of macrolides is consistent with another study [23] and is noteworthy because of increased streptococcus pneumoniae resistance to macrolides [24].

We had hypothesized that implementation of PCV after 2000 might have provided physicians with reassurance in the continued effectiveness of amoxicillin for AOM and thus lessened their tendency to prescribe broad-spectrum agents. PCV contains polysaccharides for the five serotypes of Streptococcus pneumoniae that account for most of the antibiotic resistance among pneumococci including high-level resistance to penicillin, macrolide resistance, and multidrug resistance [25]. Since its introduction, implementation of PCV has become widespread; coverage (≥ 3 doses) has increased from 41% of children aged 19 – 35 months in 2002 to 73% in 2004 [26]. Postlicensure studies have shown a decline in the prevalence of serotypes with resistance to penicillin [27-30], a reduction in treatment failures, and a reduction in frequent episodes and tube procedures [31]. Other studies have suggested that standard dose, rather than high dose, amoxicillin may once again be sufficient as first-line therapy in children who have received ≥ 3 doses of PCV [32,33]. And in France, a country with a high prevalence of antibiotic-resistant pneumococci, implementation of PCV, combined with a reduction in antibiotic use, decreased the carriage of penicillin nonsusceptible pneumococci in children with AOM [34]. One randomized trial, however, found no reduction in recurrent AOM episodes in the vaccine group [35] and a Cochrane review estimated that AOM episodes would only decrease by 6% to 7% [8].

From our results it does not appear that PCV has affected prescribing choices. Children less than 2 years of age, for instance, the group most likely to have been administered the complete schedule of PCV doses during the study period, were just as likely as older children to have received broad-spectrum antibiotics. What may be particularly concerning about these prescribing trends, in fact, is their concurrence with PCV implementation. One author has posed that resistant isolates of Streptococcus pneumoniae may reflect an adaptive response of nonvaccine serotypes to the widespread use of broad-spectrum antibiotics [36]. This may represent the type of adaptive response that has contributed to the emergence of multiresistant serotypes [36]. So, rather than providing reassurance to clinicians to continue treating AOM with amoxicillin, PCV, in combination with broad-spectrum prescribing, may actually be a contributor to antibiotic resistance. Consistent with a Swedish study which showed that more children with AOM are probably being treated at home without antibiotics [37], we also found that visits for children with AOM have decreased in recent years. In the US this trend has been attributed to the national PCV program [4,7].

We also noted that as broad-spectrum antibiotic prescribing increased, amoxicillin prescribing remain stable, despite the operant guideline for this time period recommending high dose amoxicillin as first-line therapy for most children [11]. Of note, the study period ended before the most recent guidelines on AOM [9], recommending amoxicillin/clavulanate as the initial choice for children with severe infection, would have had a significant impact on amoxicillin prescribing. Encouragingly, we found a significant decrease in the proportion of patients receiving narrow-spectrum antibiotics other than amoxicillin from 1998 to 2004. These antimicrobials have been shown to be less effective options for AOM and are no longer recommended therapy in the United States [38,39].

We found that broad-spectrum prescribing was less likely in those children with ear pain and was not associated with age or fever; factors characteristic of more severe infections that might have justified broader coverage [40,41]. Instead, significant associations were for the nonclinical factors of race, insurance status, geographical location, and ambulatory setting. Over-prescribing of antibiotics to pediatric white patients has been reported in another study [42]. High antibiotic prescribing rates have been found in the South in other studies [43,44]. And lower rates of nonrecommended (broad-spectrum) antibiotics for children with sore throats seen in emergency departments have been reported as well [42]. In contrast, general practitioners in the Netherlands were more likely to overprescribe antibiotics, according to Dutch guidelines, to younger children who were more severely ill [45]. Unfortunately, due to limitations in the data, we were unable to explore associations with other risk factors linked to the presence of bacterial species likely to be resistant to amoxicillin. These include attendance at child care and recent prescription (less than 30 days) of an antibiotic [46,47]. However, there is no reason to believe that these factors would have changed significantly over the 7 year period.

There were some aspects of the data that may limit the conclusions that can be drawn from our results. First, the NAMCS and NHAMCS survey format does not allow for direct linkage between diagnosis and medication. Even though we attempted to use a rigorous method for linking the antibiotic prescribed during a visit to an AOM diagnosis, it is possible that the antibiotics were actually prescribed for a different condition than was addressed in the visit. Second, as mentioned previously, multiple visits by the same individual are not accounted for because individual identifiers are not coded in NAMCS and NHAMCS. Lastly, we were unable to determine if high dose amoxicillin (80 to 90 mg/kg/day) was prescribed to children who received an amoxicillin prescription. High dose amoxicillin was recommended as first line treatment for AOM by a CDC consensus panel in 1999 due to concerns of drug-resistant Streptococcus pneumoniae [11]. It is possible that the rate of prescribing of high dose amoxicillin, in keeping with these guidelines, increased despite the overall stable trend in amoxicillin prescribing.

The CDC has championed national efforts to address antibiotic overuse through education of healthcare providers and the public [48]. And although overall pediatric antibiotic prescribing rates [5] and visits for AOM have decreased [4,7], it is concerning that prescribing for AOM, now known to be coupled with a high likelihood of a broad-spectrum antibiotic prescription, has not [6]. Perhaps the best means of avoiding unnecessary broad-spectrum antibiotics for AOM is to limit those children receiving any antibiotic prescription. In this regard, we support the national AOM practice guidelines that allow for the option of observation as the first-line treatment of uncomplicated AOM in children with mild disease [9]. These guidelines have been further supported by recent, randomized, controlled trials in multiple settings as well as several older metaanalyses [49-53]. Adherence to the guidelines would not only directly decrease antibiotic prescriptions for broad-spectrum and other agents, but perhaps, also indirectly, as more parents came to learn that a doctor's visit is not always necessary when their child's symptoms are mild.

## Conclusion

In conclusion, the prescribing of broad-spectrum antibiotics for AOM has increased dramatically from 1998 to 2004 and the trend occurred in the midst of the implementation of a national immunization program effective in decreasing the severity and incidence of this common pediatric infection. Physicians have increased their prescribing of broad-spectrum antibiotics for AOM without correlation to clinical factors such as age or severity of illness. If primary care physicians decide antibiotics are needed for AOM, prescribing amoxicillin as initial therapy is recommended for most children, especially with the reassurance of its effectiveness provided by PCV.

## Competing interests

The authors declare that they have no competing interests.

## Authors' contributions

AC conceived and designed the study and participated in the analysis and writing. MH participated in the design and analysis. AG coordinated and participated in the analysis and writing of the study. All authors read and approved the final manuscript.

## Pre-publication history

The pre-publication history for this paper can be accessed here:


